# Organization enhances collective vigilance in the hovering guards of *Tetragonisca angustula* bees

**DOI:** 10.1093/beheco/ary086

**Published:** 2018-06-12

**Authors:** Kyle Shackleton, Denise A Alves, Francis L W Ratnieks

**Affiliations:** 1 Laboratory of Apiculture and Social Insects (LASI), School of Life Sciences, University of Sussex, Brighton, UK; 2 Departamento de Entomologia e Acarologia, Escola Superior de Agricultura “Luiz de Queiroz”, Universidade de São Paulo, Av. Pádua Dias, Piracicaba, São Paulo, Brazil

**Keywords:** coordinated vigilance, defense, self-organization, nest, social insect, stingless bee

## Abstract

One benefit of group living is vigilance against predators. Previous studies have investigated the group size effect, where individual vigilance decreases as group size increases without reducing the overall ability of the group to detect predators. However, there has been comparatively little research on whether the positioning of individuals can improve the collective vigilance of the group. We studied the coordination of vigilance and its effect on predator detection in the eusocial bee *Tetragonisca angustula*. Nests are defended by hovering guards that detect and intercept intruders before they reach the nest entrance, in addition to those that stand upon it. We show that hovering guards are positioned nonrandomly, with a strong tendency for equal numbers on both sides of the entrance. This organization increases the collective vigilance of the guard group, as groups distributed in an even ratio, either side of the entrance, have a greater collective field of view than groups that deviate from an even ratio. Finally, we use a bioassay to show that when guards are on both sides of the entrance, their ability to detect intruders before they reach the entrance increases. Overall, our results provide strong evidence that vigilance is coordinated and that this improves nest defense. Although other group-living animals are often selfish in their individual vigilance behaviors and face competing time constraints such as foraging, the altruistic nature of eusocial insect workers has probably facilitated the evolution of coordinated vigilance, as documented here in *T. angustula*.

## Teaser

Many animals display vigilance behaviors in order to detect predators. We found that the guards of a social bee coordinate their vigilance, which increases nest defense. Guards hover and distribute themselves evenly around the nest entrance. This increases the group’s field of view and ability to detect predators. We discuss how and why this organized pattern might arise.

## INTRODUCTION

Vigilance against predators is one potential benefit of group living, as it can increase predator detection and individual survival (Pulliam 1973; Krebs and Davies 1993; Cresswell 1994; Beauchamp 2017). An increase in group size also leads to a reduction in the time that individual group members spend being vigilant (Bertram 1980; Elgar and Caterall 1981; Lima 1995). This group size effect is commonly explained by either the many-eyes hypothesis, where the proportion of time at least one individual is scanning increases (Bertram 1980; Lima 1995; Fairbanks and Donson 2007), or the dilution effect whereby each individual is at lower risk of being targeted by a predator (Hamilton 1971; Dehn 1990; Roberts 1996). By spending less time vigilant, individuals can dedicate more time to foraging or other activities that enhance fitness (Elgar and Caterall 1981). For example, Lima (1995) found that dark-eyed juncos, *Junco hyemalis*, consumed food items over 50% faster as group size increased from 1 to 6.

The collective vigilance of a group would be increased if group members also coordinated their vigilance efforts, such as by looking in different directions. Alternatively, coordinated vigilance may be organized so that some individuals focus on vigilance allowing others to focus on foraging. Although models predict benefits of coordination to collective vigilance (Bednekoff and Lima 1998; Ferriere et al. 1999), such behavior is rarely observed in nature (Ward 1985; Pays et al. 2007). This may be because individuals are selfish (Hamilton 1971) or that the need to monitor the vigilance status of neighbors is itself costly and provides only marginal benefits over noncoordinated vigilance (Ward 1985; Rodríguez-Gironés and Vasquez 2002). Where coordinated vigilance has been observed, it usually involves a sentinel system of only 1 or 2 vigilant individuals (meerkats, Clutton-Brock et al. 1999; cranes, Ge et al. 2011; rabbitfish, Brandl and Bellwood 2015). However, how vigilant individuals position themselves relative to each other and how this affects collective vigilance have received less attention than the effect of group size.

Vigilance in social insects differs from most vertebrate examples in that, rather than fleeing from predators, vigilance may improve the defense of a fixed location, the nest. The nest contains reproductive individuals, offspring (brood), and food stores, such that its defense provides large fitness benefits. Early detection of predators is important for social insects because the first predators to arrive are often scouts of other social-insect colonies that can recruit nestmates for a mass attack (Blum et al. 1970; Michener 1974; Ono et al. 1995). Detecting and disabling these scouts is, therefore, critical for colony survival. The second important distinction is that social insects often possess dedicated defenders (guards), which sometimes have morphological specializations and are not constrained by the need to forage or reproduce. Rather, time and effort are traded-off at the colony level through division of labor, with workers allocated among different tasks.

**Figure 1:**
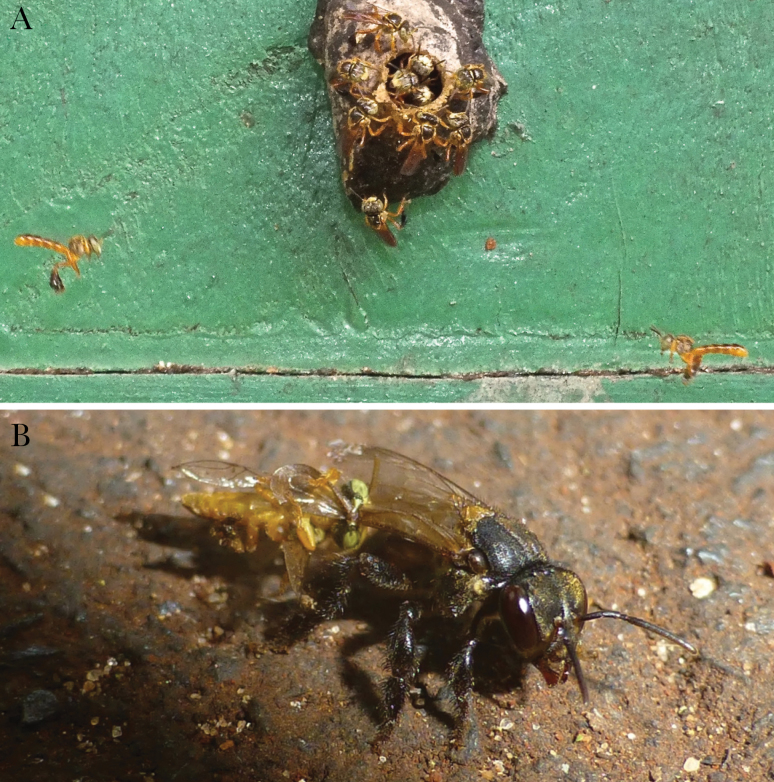
(A) Hovering guards of the stingless bee *Tetragonisca angustula* at a nest entrance in São Paulo State, Brazil. Hovering guards are positioned one on each side of the entrance tube to form a corridor through which incoming bees must pass. Standing guards can be seen in and around the entrance. (B) *T. angustula* guard (left) fighting with *Lestrimelitta limao* robber bee (right).

The stingless bee *Tetragonisca angustula* (Apidae: Meliponini) presents an excellent opportunity to study the group-level coordination of vigilance. In addition to guards that stand at the nest entrance, which is normal in social insects, *T. angustula* colonies also have guards that hover near the entrance (Grüter et al. 2011). To date, hovering guards are only known in *T. angustula* and the closely related *T. fiebrigi* (Grüter C, personal communication). Hovering guards are normally positioned to the left and right of the entrance and face inwards to form a corridor through which most bees entering the nest must pass (Figure 1A, Wittman 1985). Guards inspect incomers, intercept nonnestmates and wrestle them to the ground (Wittman et al. 1985). In agreement with studies of vigilance in vertebrates, larger groups of hovering guards are better at detecting intruders (van Zweden et al. 2011). Furthermore, *T. angustula* guards are morphologically specialized, being the first described and most prominent example of a soldier caste within the eusocial bees (Grüter et al. 2012; Grüter et al. 2017). The main natural enemy of *T. angustula* is the obligate robber bee *Lestrimelitta limao* (Figure 1B), which probably drove the evolution of the soldier caste (Grüter et al. 2017) and whose local density influences colony investment in defense (Segers et al. 2016).

We investigated how *T. angustula* hovering guards are positioned relative to each other and how this affects vigilance and predator detection. Guards typically hover on either side of the entrance tube, looking inwards and pointing left or right (Figure 1A). As a result, a guard facing left of the entrance will have a more limited view of the right of the entrance and *vice versa*. To enhance their collective vigilance, we predict that guard groups should have individuals facing both directions. Our first aim was to establish whether hovering guards were positioned more evenly, left and right of the entrance, than expected if they positioned themselves randomly. We then compared vigilance of even versus skewed left–right distributions of hovering guards. Finally, we investigated the effect of guard distribution on the ability to detect predators using a bioassay to simulate an attack by *L. limao* robber bees.

## METHODS

### Study site and colonies

The study was carried out at the Department of Entomology and Acarology at the University of São Paulo, Piracicaba, Brazil. Data were collected in March 2017 from 08:30 to 16:30 in good weather conditions when colonies were active. We studied 15 colonies of *T. angustula* stingless bees (Meliponini: Apidae), including 4 wild colonies and 11 in hives. This species is considered mildly aggressive amongst the stingless bees (Shackleton et al. 2015), but is especially aggressive towards the robber bee *L. limao* (Sakagami et al. 1993; Grüter et al. 2012). All colonies had built their characteristic entrance tube and had standing guards positioned at the nest entrance (Figure 1A). At the end of each day of data collection, we removed the hovering guards from each colony entrance to minimize any pseudoreplication that might arise from gathering data from the same individuals across days.

### Distribution of hovering guards

We made a count of the hovering guards at the nest entrance of each colony, recording the number to the left and right of the entrance for groups of 2 or more (*n* = 66, 46, and 22 for 2, 3, and 4 guards, respectively). For each guard number (2, 3, or 4), the frequency with which guards were distributed in all possible arrangements, left versus right, was then compared with the expected random distribution. That is, if the probability of each individual being positioned to the left or the right was 0.5. Expected distributions were calculated using the binomial theorem with the formula (*p* + *q*)^*n*^, where *p* is the probability of a bee being observed on the left (0.5), *q* (= 1 − *p*) is the probability of a bee being observed on the right (0.5) and *n* is the total number of bees. For example, for 2 bees the formula (*p* + *q*)^2^ can be expanded to *p*^2^ + 2*pq* + *q*^2^. This equates to probabilities of 0.5^2^ = 0.25, 2 bees left; 2 × 0.5 × 0.5 = 0.5, 1 bee either side; and 0.5^2^ = 0.25, 2 bees right. We then used chi-square tests to compare our observed and expected distributions.

### Vigilance of hovering guards

We investigated the effect of guard number and arrangement (left vs. right) on the vigilance of individual hovering guards and on the collective vigilance of the group. Most previous studies have measured vigilance by the degree of scanning, in which an individual raises its head and surveys its environment (Elgar et al. 1989), or by the time taken to consume food items (Lima 1995). The small size of *T. angustula* makes measuring head movements in the field impractical. Furthermore, guards do not forage, spend all of their time guarding, and are presumably always “scanning.” However, guards often change orientation as they hover. We therefore quantified body rotation as a measure of individual vigilance (Ward et al. 2011).

We video-recorded the nest entrance from 2 directions: directly above the entrance to measure lateral rotation, then directly facing the entrance hole to measure longitudinal rotation. For each video, we counted the number of guards present and extracted 5 still images 10-s apart. We imported the images into ImageJ (Schneider et al. 2012) and used the angle tool to measure the angle of each bee relative to the entrance. From these 5 angles, we calculated the range of rotation as a measure of variation in the orientation of each bee. This range does not represent the total field of view of the bee, because the eyes are situated on the side of the head providing a wider view of the environment than human vision. However, the visual field of a bee contains an area of dead space (or blind spot) at the center of the posterior hemisphere of the head (Seidl and Kaiser 1981), and the resolution is lower towards the posterior of the eye (Land and Nilsson 2012). Rather, the range represents the degree to which each bee moved and so increased its view of the environment.

Preliminary observations of hovering guards indicated only minor rotation in the longitudinal plane. That is, there was little tilting of the head or body up and down, mean range ± SD = 7.63 ± 3.08°, *n* = 10. We observed far greater rotation in the lateral plane, pivoting side to side, 33.24 ± 13.20°, *n* = 10. Therefore, we focused on scanning behavior in the lateral plane and recorded 33, 38, 24, and 24 individual guards for groups of 1, 2, 3, and 4 hovering guards, respectively.

To quantify the collective vigilance of the guard group, we calculated the total angle covered by each of the above groups (*n* = 33, 19, 8, and 6 groups for groups of 1, 2, 3, and 4 guards, respectively). The angles of all individual guards within a group were summed minus any overlap in ranges. For example, if 2 bees each cover a range of 90° with no overlap in field of view, then the collective vigilance of the group is 90 + 90 = 180°. If bee 2 bees both cover a range of 90° but overlap in their field of view by 20°, then the collective vigilance of the group is 90 + 90 – 20 = 160°.

To test for the effect of guard arrangement on individual and collective vigilance, we calculated a measure of deviation from an even ratio of guards defined as:

$$2\left(\left(\frac{L}{L+R}\right)-0.5\right)$$

where *L* is the number of hovering guards on the left and *R* the number on the right. We converted the values to the absolute values, to give a range between 0 (evenness) and 1 (all bees on one side of the entrance). For example, an arrangement of 2 bees left and 1 right would yield a deviation of 2 × ((2 / 3) − 0.5) = 0.33.

To analyze these data, we fitted 2 mixed-effects models with Gaussian distributions, 1 with the individual range of lateral rotation as the response variable, and the other with the total angle covered by the guard group as the response variable. In each case, we fitted guard number (a factor with levels 1, 2, 3, and 4) and deviation (a continuous variable ranging from 0 to 1) as explanatory variables and colony as a random effect. We performed post hoc multiple comparisons where guard number was significant.

### Detection of a model predator

To investigate the effect of guard orientation on the ability of a colony to detect predators, we studied the simplest configurations of hovering guards; a single guard (*n* = 58) and 2 guards (*n* = 40), one on either side of the entrance. As well as being common (see Results), these simple configurations allowed us to address 2 questions: First, for a single guard, what is the probability of the guard detecting an intruder when it approaches from the guard’s front versus behind? Second, for 2 guards and an intruder approaching perpendicular to the entrance such that it is directly in front of 1 guard and behind the other, which guard detects the intruder first?

We simulated the attacks of *L. limao* robber bees using a dummy bee made of black modeling clay (10 × 3 × 3 mm), following van Zweden et al. (2011). The dummy was treated with citral (Sigma Aldrich, Stenheim, Germany) a major component of *L. limao* mandibular glands and known to elicit aggressive defensive responses in *T. angustula* (Wittman 1990; van Zweden et al. 2011). We suspended the dummy from a wooden pole via a thread (diameter = 0.3 mm) and introduced it perpendicular to the colony entrance, directly in front of and/or behind the hovering guards, not head-on to the entrance as in van Zweden et al. (2011). To elicit an attack from *T. angustula*, we began moving the dummy towards the colony entrance from an initial distance of 20 cm at a constant rate of 1 cm s^−1^ until an attack occurred. An attack was defined as a hovering guard flying directly towards and grasping the dummy, at which point the trial was terminated. If the dummy reached the entrance without receiving an attack from the bees then the trial was terminated. Each trial used a fresh dummy. For the assay using a single guard, we analyzed the data using a mixed-effects model fitting probability of attack as the response variable, attack direction as the explanatory variable, colony as a random effect, and a binomial error structure. For the assay using 2 guards, we compared the number of attacks to the dummy from the guard facing the dummy (front) versus the guard facing away from the dummy (behind) using a chi-square test.

### General statistical methods

All statistical analyses were conducted using R version 3.3.2 (R Core Team 2016), including the packages lme4 and lmerTest for mixed-effects models (Bates et al. 2015; Kuznetsova et al. 2017) and lsmeans for post hoc tests (Lenth 2016). *P* values and test statistics are reported from the ANOVA function of the lmerTest package.

## RESULTS

### Distribution of hovering guards

In total, we made 287 observations of our 15 nests. The most common number of hovering guards present was 1 (23.7%) followed by 2 (23%), 3 (16.0%), and 4 (7.7%). Zero guards were present in 12.9% of observations and in the remainder there were ≥5 guards present (16.7%), see Supplementary Figure. We found no overall bias for guards to be positioned on either the left or right of the entrance, 406 left versus 411 right (chi-square test χ^2^ = 0.031, *P* = 0.861, DF = 1). This validated our random model (see Methods), in which *p* = *q* = 0.5 and allowed us to combine inverse ratios. For example, in a group of 4 guards, the counts left:right of 1:3 and 3:1 were pooled.

**Figure 2:**
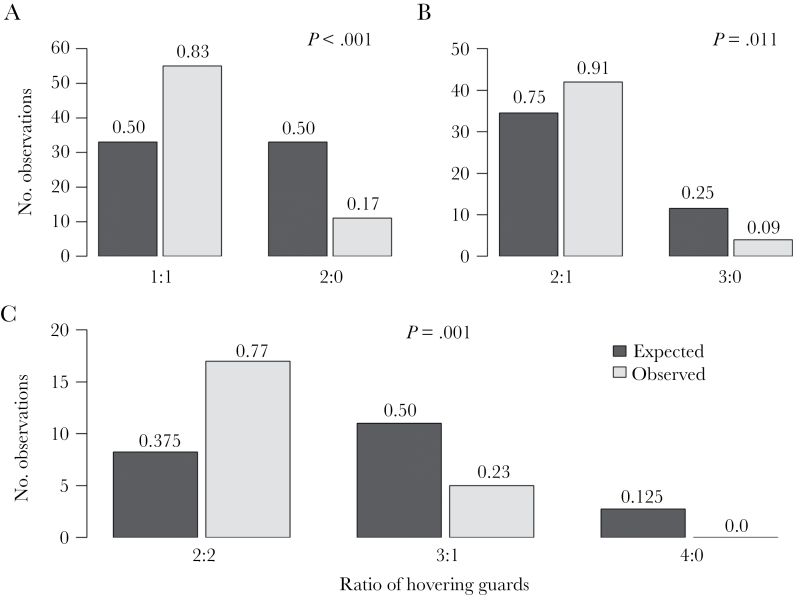
Observed and expected numbers of *Tetragonisca angustula* hovering guards for arrangements of (A) 2 guards (*n* = 66 guard groups), (B) 3 guards (*n* = 46), and (C) 4 guards (*n* = 22). Symmetrical arrangements are combined, for example, 2:0 with 0:2. Numbers above bars indicate proportions and *P* values indicate significant differences in observed versus expected numbers.

Hovering guards were significantly more likely to be distributed evenly on both sides of the entrance than randomly. This was true for all arrangements for which our sample size was sufficient; 2 (χ^2^ = 29.333, *P* < 0.001, DF = 1, *n* = 66, Figure 2A), 3 (χ^2^ = 6.522, *P* = 0.011, DF =1, *n* = 46, Figure 2B) and 4 guards (χ^2^ = 15.303, *P* < 0.001, DF = 2, *n* = 22, Figure 2C). The sample size for arrangements of ≥5 guards was too small for analysis.

### Vigilance of hovering guards

**Figure 3:**
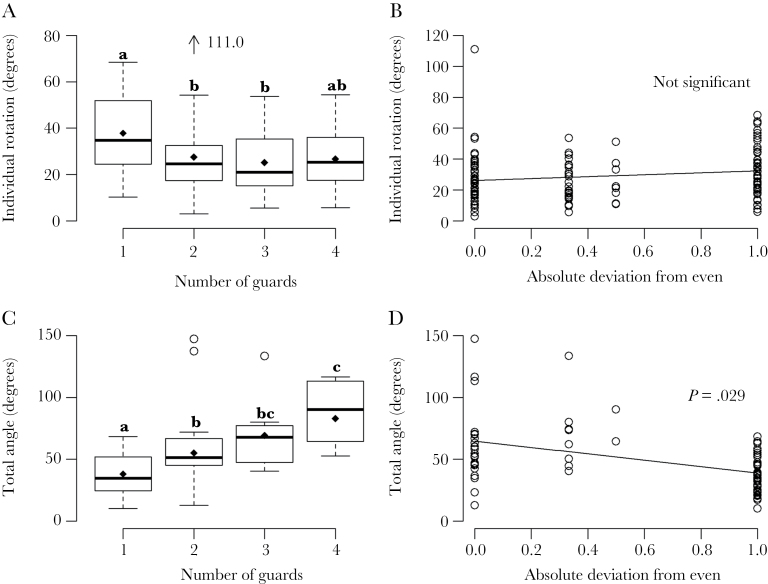
Vigilance in hovering guards of *Tetragonisca angustula* bees, measured as range of lateral rotation. (A) and (B) show the effect on individual vigilance of guard number and deviation in guard ratio from even (left vs. right). (C) and (D) show the effect on collective vigilance of guard number and deviation. Range calculated from the maximum minus minimum angle relative to the nest entrance from 5 snapshots of the position of a guard. A deviation of 0 indicates an even ratio of guards, whereas 1 indicates that all guards were on one side of the entrance. Diamonds indicate means. Whiskers are 1.5× the interquartile range. Letters indicate significance following post hoc tests, circles and arrows indicate outliers. For groups of 1, 2, 3, and 4 guards, *n* = 33, 38, 24, and 24 individual guards and 33, 19, 8, and 6 guards groups, respectively.

Guard number had a significant effect on the lateral scanning behavior of individual guards (Figure 3A, mixed-effects model, *F* = 3.228, *P* = 0.025, DF = 3). Lone guards rotated laterally 42% more than guards in groups of 2 or more (37.8 ± 15.7° compared with 26.6 ± 15.0°). Post hoc multiple comparisons found significant differences in rotation between lone guards (*n* = 33) and those in groups of either 2 (*P* = 0.015, *n* = 38) or 3 (*P* = 0.013, *n* = 24). There was no significant difference in rotation between 1 and 4 guards (*P* = 0.092), perhaps due to the low sample size for 4 guards (*n* = 24) providing insufficient statistical power, and there were no significant differences among guard numbers greater than 2 (*P* > 0.05 in all cases). The arrangement of guards (their left–right ratio) had no effect on the rotation of individual guards (Figure 3B, mixed-effects model, *F* = 0.461, DF = 1, *P* = 0.5476).

Collective vigilance in hovering guards increased significantly from 1 to 4 guards (Figure 3C, mixed-effects model, χ^2^ = 26.944, DF = 3, *P* < 0.001). Guards in groups of 4 (*n* = 6) had a collective range of 82.9 ± 25.4° compared with 37.8 ± 15.7° for lone guards (*n* = 33), more than double. This was largely due to having guards on both sides of the entrance rather than simply having more guards, because each additional guard on the same side of the entrance overlaps successively more in its visual range with those already present. Furthermore, guard groups arranged in a more even ratio had a significantly greater collective visual range than groups that deviated from even (Figure 3D, mixed-effects model, *F* = 4.977, DF = 1, *P* = 0.029).

### Detection of a model predator

In contrast to van Zweden et al. (2011), *T. angustula* hovering guards did not always attack the dummy intruder, 49% in this study versus 100% in van Zweden et al. (2011). This may be because of differences in our methodology, as we purposefully used colonies with a small number of guards (1 or 2) and introduced the intruder at more difficult angles to detect, that is, from the side rather than head on. The dummy was attacked 22/58 times when presented to a single guard (38%) compared with 40/69 times for 2 guards, one on either side of the entrance (58%). This difference was significant (proportion test, χ^2^ = 9.51, DF = 1, *P* = 0.002).

**Figure 4:**
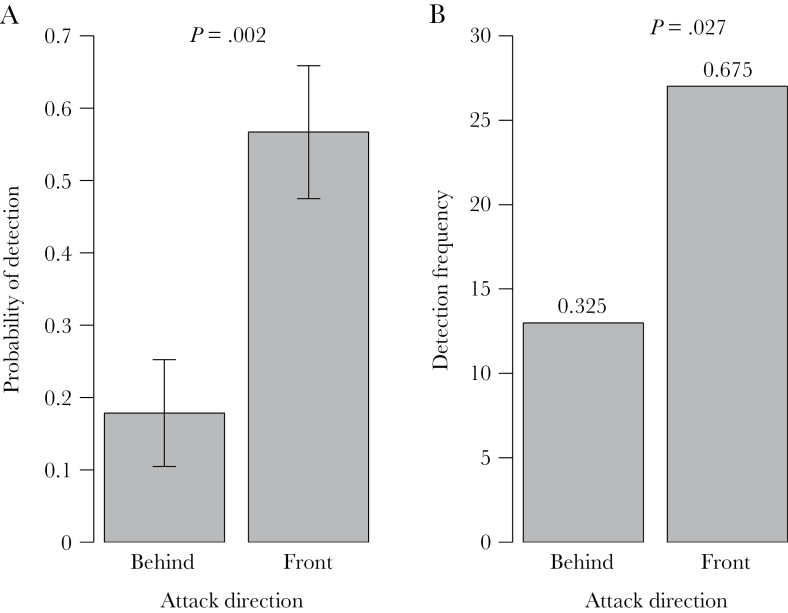
Ability of hovering guards of *Tetragonisca angustula* to detect predator models approaching from directions perpendicular to the colony entrance when the number of hovering guards is (A) 1 and (B) 2, one on either side of the entrance. When one guard was present, a dummy predator was introduced either in front of or behind the guard, and the probability of the guard attacking the dummy was recorded (*n* = 22 attacks from 58 trials). When two guards were present, the attacking bee was recorded as either the one facing (front) or not facing (behind) the dummy (*n* = 40 attacks from 69 trials). *P* values indicate significance, numbers above bars indicate proportions.

Single hovering guards presented with a dummy intruder were 3 times more likely to detect and attack it before it reached the nest entrance when it approached from the front versus from behind the guard (Figure 4A). This difference was significant (mixed-effects model, χ^2^ = 9.52, *P* = 0.002, DF = 1, *n* = 58). When we presented the dummy to 2 guards, one on either side of the entrance, the dummy was twice as likely to be attacked by the guard facing the dummy, as opposed to the guard facing away (Figure 4B, chi-square test, χ^2^ = 4.900, DF = 1, *P* = 0.027, *n* = 40). This result is especially striking, given that the guard facing the dummy was actually further from the dummy. Together, these results confirm that guards are better able to detect intruders that approach from the front rather than behind.

## DISCUSSION

Our results show that multiple hovering guards of *T. angustula* coordinate themselves in a way that improves the collective vigilance of the group. Hovering guards were distributed more evenly, left versus right, than would be expected if each individual was positioned at random. This effect was significant in each of 3 situations: 2, 3, and 4 guards, providing strong evidence for colony-level adaptive organization. The effect was weaker when 3 guards were present. However, this was likely because the expected frequency of guards in the most even ratio (2:1) was 75%, meaning that the maximum possible effect size in the direction of evenness was only 25% (Figure 2B) versus 50% (50% expected) when 2 guards were present (Figure 2A).

The coordination of hovering guards into an even ratio increased the collective vigilance of the group, but did not have an effect at the individual level (Figure 3B,D). Meanwhile, an increase in group size caused a decrease in individual vigilance but an increase in collective vigilance, consistent with the group-size effect (Figure 3A,C). The decrease in individual rotation may be beneficial, if rotation somehow reduces the quality of vision of the guard and, presumably, the guard saves a small amount of energy. The individual response to group size may be adaptive, resulting from an awareness that other hovering guards are present. Alternatively, the increased level of rotation in small groups may be because every guard has to inspect incoming bees, whereas in large groups, some inspect while some remain in position and so rotate less. The collective response to group size was greatest between 1 and 2 guards and was enhanced by coordination, because the second guard was typically on the opposite side to the first, which generally doubled the total field of view.

Hovering guards seldom face outwards from the nest entrance, which would seemingly limit the group’s collective view of the environment. However, the compound eyes of *T. angustula* extend to the side of the head (see Grüter et al. 2012), allowing the bee to see outwards even when its body is perpendicular to the nest entrance. Coupled with the generally poor visual acuity of the insect compound eye (Mallock 1894; Kirschfield 1976; Snyder 1977; Land 1997), this suggests that the addition of guards facing outwards would not greatly increase predator detection. The positioning of hovering guards to face a flight corridor has the additional function of increasing the ability of guards to intercept intruders flying towards the entrance (Wittman 1985).

Guards facing in the direction of attack were better able to detect intruders, as shown in our 2 complementary bioassays. Lone guards were 3 times as likely to detect a dummy robber bee when it approached from the front, rather than the rear. When there were 2 hovering guards, the guard facing the intruder was twice as likely to initiate an attack as the guard facing away. This second result is all the more powerful because the guard facing the model predator was always the further from it of the two. The diffusive nature of larger guard groups may lead to the breakdown of this rule, because an intruder will have to bypass several guards facing away before it encounters a guard facing towards it. The direct defensive benefits of coordinated over noncoordinated vigilance is a topic for further study. In particular, it would be valuable to investigate whether coordinated vigilance in *T. angustula* increases the ability of a colony to defend against the robber bee *L. limao*, which is probably the most important enemy of *T. angustula* (Segers et al. 2016; Grüter et al. 2017). Furthermore, is coordinated vigilance more efficient than noncoordinated vigilance? For example, do 2 hovering guards in an even left–right ratio may have greater collective vigilance than 3 guards that all hover on the same side of the entrance, meaning that fewer guards are needed?

Coordinated vigilance in *T. angustula* is presumably adaptive in the context of the behavior and strategy employed by *L. limao* robber bees, especially scouts, when approaching a *T. angustula* nest entrance. If robber bees approach from the side then the coordination of vigilance is clearly of value, as shown by our bioassays. However, if robber bees approach from the front then we would not expect coordination to be more effective than if guards were positioned at random. If robber bees do not employ any positional strategy and instead attack from a random direction, then the coordination of vigilance will be of use at least some of the time, and there is presumably little additional cost of coordinated versus uncoordinated vigilance. Unfortunately, to witness the beginning of a raid, where robber bee scouts first find the host nest, is extremely rare (von Zuben 2012; Grüter C, personal communication), and we have not ourselves witnessed the initial stages of an attack. It would therefore be of great value to observe the initial stages of a raid and to study the response of hovering guards.

The benefits of coordinated vigilance relative to the more established role of group size remain unknown. Although we studied groups of 1–4 hovering guards, the number may exceed 15 (van Zweden et al. 2011). We predict that as group size increases, the importance of coordination relative to group size will diminish for 2 reasons: first, because coordination will become more difficult, analogous to the costs of monitoring other group members proposed by Ward (1985); second, with many guards even a random configuration would likely cover all directions. Furthermore, as guard number increases, we expect collective vigilance (Figure 3C) to plateau as it approaches the limit of 360°. However, higher guard number could still increase collective vigilance through the occupation of a greater area (van Zweden et al. 2011). There would also be defensive benefits unrelated to vigilance, in particular, the ability to fight, harass, or confuse predators should continue to increase with group size (Shields 1984; Landeau and Terborgh 1986; Shackleton et al. 2015). Indeed, there are also several guards that stand at the nest entrance, ready to attack any threats once they are detected.

How do hovering guards achieve an even left–right distribution? We hypothesize that the pattern is self-organized, which is a common mechanism in insect societies, including nest defense (Bonabeau et al. 1997; Millor et al. 1999; Boomsma and Franks 2006). There is also evidence that self-organization works in conjunction with group size to produce greater collective vigilance in fish shoals (Ward et al. 2011). The pattern in *T. angustula* could arise through individual guards reacting to their own local environment and experience, with the application of 2 simple rules: first, if a guard detects another guard on the same side of the entrance as itself, then its propensity to switch sides increases; second, if after switching a guard detects another guard on the same side as itself, it remains for some time before moving, in order to prevent continuous switching. Alternatively, a guard may react to the absence of guards on its side or the guard state on the opposite side to itself. There is some evidence that bees can count, at least up to 4 (Chittka and Geiger 1995; Dacke and Srinivasan 2008), which might also be used in distributing hovering guards into an even ratio.

In contrast to individuals in an ungulate herd, bird flock, or fish shoal, social-insect guards should always be vigilant. Because worker fitness is tied more closely to the colony than personal safety, guard groups should be free from the limitations of the selfish herd (Hamilton 1971), which may prevent the emergence of collective vigilance arising from the cooperation of unrelated individuals. In selfish herding, unrelated individuals should strive for the safe positions with little regard for the interests of their neighbors (Hamilton 1971). The study of organized patterns in animals with high intra-group relatedness (e.g. Santema and Clutton-Brock 2013) may reveal new rules governing vigilance and the benefits of group living in general.

## SUPPLEMENTARY MATERIAL

Supplementary data are available at *Behavioral Ecology* online.

Supplementary Figure S1

Supplementary Figure Legends

## FUNDING

K.S.’s PhD is jointly funded by the National Environment Research Council (Grant Number: NE/K501347/1) and the University of Sussex School of Life Sciences. F.L.W.R. received travel funds from the São Paulo Research Foundation (FAPESP, Grant Number: 2016/22861–6) and Santander Bank. D.A.A. is funded by PNPD/CAPES (Programa Nacional de Pós-Doutorado da Coordenação de Aperfeiçoamento de Pessoal de Nível Superior).

## 

Data accessibility: Analyses reported in this article can be reproduced using the data provided by Shackleton et al. (2018).
