# Supplementary figures and images for: Organization enhances collective vigilance in the hovering guards of Tetragonisca angustula bees
Source: Behav Ecol. 2018 Jun 12;29(5):1105–12. doi: 10.1093/beheco/ary086 (PMC6129946; doi:10.1093/beheco/ary086)

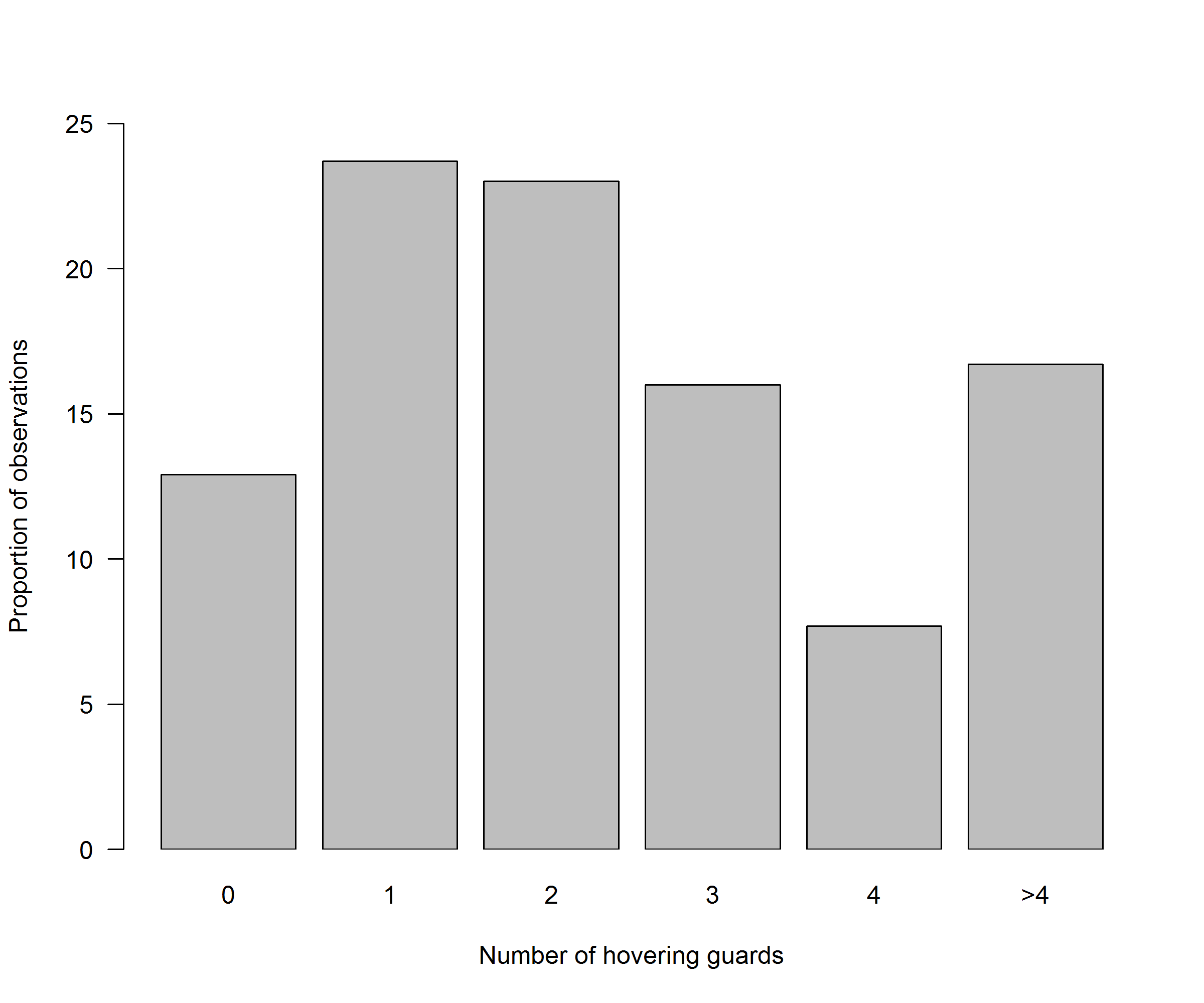

Supplement: Supplementary Figure S1 [file ary086_suppl_figure_s1.png]
