# Supplementary material for: Organization enhances collective vigilance in the hovering guards of Tetragonisca angustula bees
Source: Behav Ecol. 2018 Jun 12;29(5):1105–12. doi: 10.1093/beheco/ary086 (PMC6129946; doi:10.1093/beheco/ary086)
Supplement: Supplementary Figure Legends [file ary086_suppl_supplementary_figure_legends.docx]

**Supplementary Figure legends**

Supplementary figure: The frequency distribution of the number of hovering guards in guard groups of *Tetragonisca angustula*.
